# Modeling dynamic behavior of dielectric elastomer muscle for robotic applications

**DOI:** 10.3389/fbioe.2023.1006346

**Published:** 2023-02-10

**Authors:** Seung Mo Jeong, Heeju Mun, Sungryul Yun, Ki-Uk Kyung

**Affiliations:** ^1^ Human-Robot Interaction Laboratory, Department of Mechanical Engineering, Korea Advanced Institute of Science and Technology, Daejeon, Republic of Korea; ^2^ Tangible Interface Creative Research Section, Electronics and Telecommunications Research Institute (ETRI), Daejeon, Republic of Korea

**Keywords:** dielectric elastomer, soft actuator, modeling, dynamic actuation, simulation

## Abstract

Recently, as a strong candidate for artificial muscle, dielectric elastomer actuators (DEAs) have been given the spotlight due to their attractive benefits from fast, large, and reversible electrically-controllable actuation in ultra-lightweight structures. Meanwhile, for practical use in mechanical systems such as robotic manipulators, the DEAs are facing challenges in their non-linear response, time-varying strain, and low load-bearing capability due to their soft viscoelastic nature. Moreover, the presence of an interrelation among the time-varying viscoelasticity, dielectric, and conductive relaxations causes difficulty in the estimation of their actuation performance. Although a rolled configuration of a multilayer stack DEA opens up a promising route to enhance mechanical properties, the use of multiple electromechanical elements inevitably causes the estimation of the actuation response to be more complex. In this paper, together with widely used strategies to construct DE muscles, we introduce adoptable models that have been developed to estimate their electro-mechanical response. Moreover, we propose a new model that combines both non-linear and time-dependent energy-based modeling theories for predicting the long-term electro-mechanical dynamic response of the DE muscle. We verified that the model could accurately estimate the long-term dynamic response for as long as 20 min only with small errors as compared with experimental results. Finally, we present future perspectives and challenges with respect to the performance and modeling of the DE muscles for their practical use in various applications including robotics, haptics, and collaborative devices.

## 1 Introduction

Artificial muscles have been given great attention as the next-generation actuators for robotics and collaborative systems owing to their versatility in motion and inherent flexibility enabling the robots to be suitable for use in a safe working environment. As one of the promising candidates for artificial muscles, dielectric elastomer actuators (DEAs) have been intensively studied (Madden, 2011). Generally, the DEA is composed of a DE membrane and a couple of thin compliant electrodes. These electrodes are established on both surfaces of the DE membrane through various methods, including brushing, spraying, and sputtering. When an electrical voltage is applied across the compliant electrode, the DEA, pre-stretched and clamped onto a rigid frame, is compressed by electrostatic force in the thickness direction and simultaneously expands to the in-plane direction (Pelrine and Kornbluh, 2011). The electrically-induced actuation performance is scalable by following a simple electrostatic model, which can be expressed by the equation below.

$$\begin{array}{c} p=\varepsilon_{0}\varepsilon_{r}{\left(\frac{V}{z}\right)}^{2} \end{array}$$ (1)

where p is the effective pressure, $\varepsilon_{0}$ is the permittivity of free space, $\varepsilon_{r}$ is the relative permittivity of the dielectric elastomer, *V* is the voltage input, and *z* is the thickness. Since the relative permittivity is one of the crucial parameters to determine the achievable area strain of the DEAs, researchers have adopted diverse dielectric materials including acrylic elastomers, silicone elastomers, and polyurethanes for the DEAs (Kornbluh, 2011; Madden, 2011; Youn et al., 2020).

Due to the attractive benefits from large strain actuation, high energy density, and mechanical resilience coming from a highly flexible structure, the DEAs have been exploited for various applications such as haptic devices, soft grippers, wearable interfaces, and locomotion (Frediani et al., 2014; Yun et al., 2019; Youn et al., 2020; Chen et al., 2021; Hwang et al., 2021; Youn et al., 2021). For better usability in these applications, the integration of stiff frame, fiber, fluid, and pneumatics into a dielectric elastomer has been also attempted to improve the actuation performance of the DEAs (Huang et al., 2012; Zhang C et al., 2015; Liu et al., 2017; Hau et al., 2018; Cao et al., 2020; Costa et al., 2020; Song and Cha, 2020; Guo et al., 2021; Hwang et al., 2021). Depending on the purpose, researchers have proposed DEAs with different designs such as multilayer stack, extender, bending beam, diaphragm, and rolled structures for eliciting linear elongations, out-of-plane expansions, or bending movements from the fundamental in-plane expansion behavior responding to electrical stimulation (Kornbluh, 2011).

Of these proposed configurations, we investigate rolled DEAs in detail. Rolled DEAs, as the name suggests, are fabricated by rolling dielectric elastomer films into a cylindrical shape (Pei et al., 2003). From the rolling process, multiple DE layers are created and the actuator can withstand higher load and more actuation cycles compared to single-layer DEAs (Youn et al., 2020). When a spring core is embedded in the fabrication stage, the configuration is termed the spring-rolled DEA. Owing to the use of the compressed spring structure enabling axial stretching *via* a spring being restored from its compressed state, spring-rolled DEA could produce electrically controllable and linear tensile stroke while maintaining its structure without the need for an external frame or clamping mechanism (Pei et al., 2004).

However, despite the evolution of DEAs with various aspects, difficulty in their fine control is a huge obstacle to exploiting DEAs for practical use in robotic applications. Although many models have been proposed to establish a control strategy, long-term and/or dynamic actuation responses of the DEAs rarely allow accurate estimation due to the presence of inherent non-linear and complex time-dependent viscoelastic behavior, which is interrelated with dielectric and conductive relaxations (Zhao et al., 2011; Tran et al., 2018). Adding to the complexity, the DEA is also affected by cyclic relaxations. With repeated loading and unloading in dynamic actuation, the decrease in relaxation modulus becomes less with the increasing number of loading cycles (Sahu et al., 2015; Sahu and Patra, 2016).

In this paper, studies on the rolled DEAs are reviewed with respect to fabrication methodologies and models for predicting their actuation responses. Moreover, we propose a new model integrating hyperelastic and viscoelastic models that is capable of estimating the long-term viscoelastic creep deformation as well as temporal and frequency responses of the spring-rolled DEA. Lastly, we discuss practical considerations and perspectives for implementing DE actuators in diverse application fields.

## 2 Dielectric elastomer muscle

For rolled DE configurations, commercially available 3 M VHB acrylic elastomers, as well as silicone elastomers, are widely used thanks to their soft and elastic nature, and relatively high dielectric properties (Kornbluh and Pelrine, 2011). As a compliant electrode for the DEA, carbon-based conductive materials and metallic nanowires have been mainly adopted due to their cost-effectiveness, facile coating process in large areas, and high tolerance to electrical excitation up to several kilovolts (Kovacs et al., 2008; Rajamani et al., 2008; Kiil and Benslimane, 2009).

**FIGURE 1:**
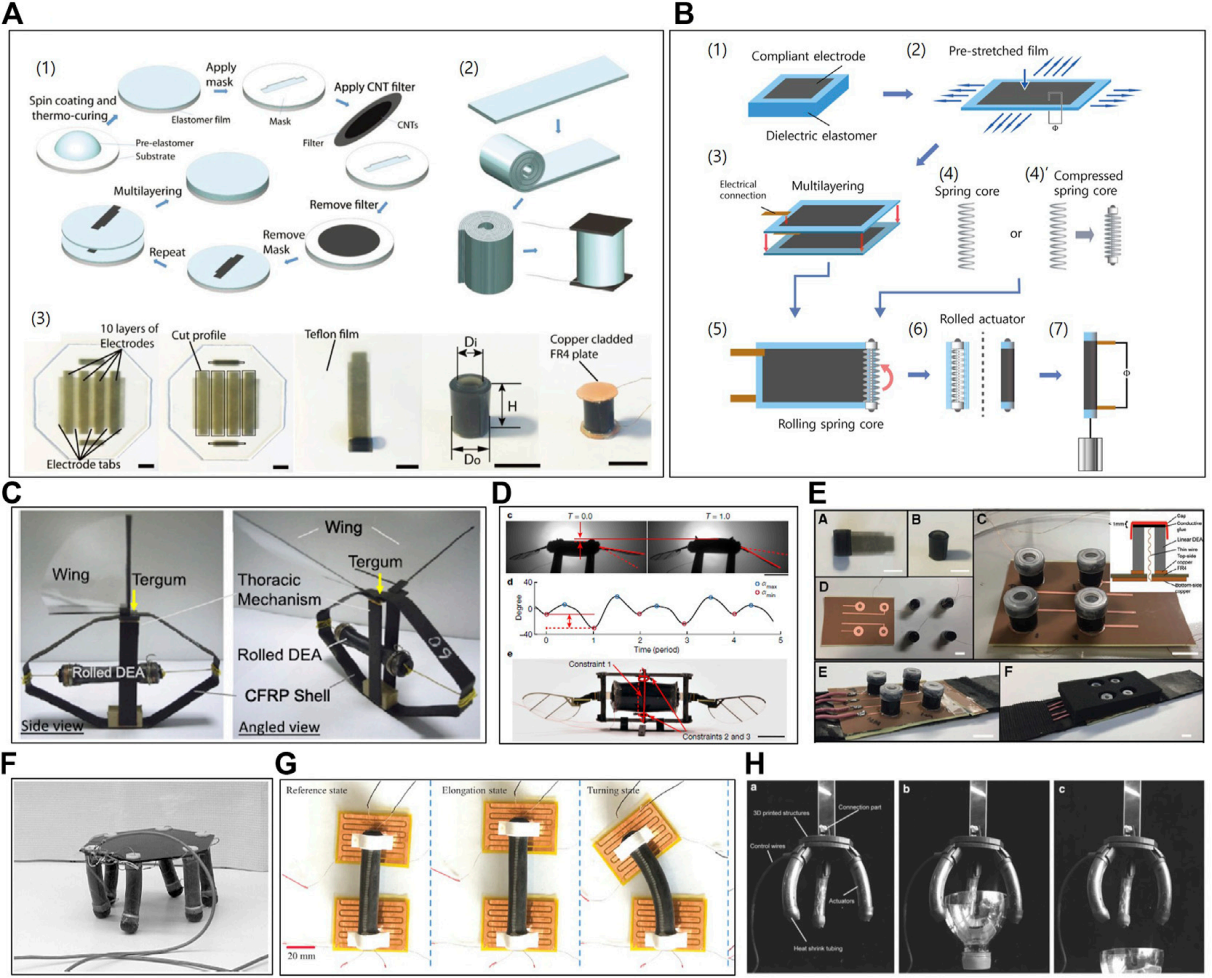
**(A)** Fabrication process of silicone-based core-free rolled DEAs. Adapted with permission from Zhao et al. (2018). Copyright 2015, John Wiley and Sons. **(B)** Fabrication process of acrylic-based spring-rolled DEAs. **(C)** Bio-inspired wing flappers driven with core-free rolled DEA. Adapted with permission from Lau et al. (2014). Copyright 2014, IOP Publishing. **(D)** Micro-flying robot powered by core-free rolled DEA. Adapted with permission from Chen et al. (2019). Copyright 2019, Springer Nature. **(E)** Wearable soft haptic communicator based on core-free rolled DEA. Adapted with permission from Zhao et al. (2020). Copyright 2020, Mary Ann Liebert, Inc. **(F)** MERbot, with spring-rolled DEAs as its legs. Adapted with permission from Pei et al. (2003). Copyright 2003, SPIE. **(G)** Locomotion robot driven by spring-rolled DEA and electroadhesive feet. Adapted with permission from Guo et al. (2022). Copyright 2022, Elsevier. **(H)** Gripper with three spring-rolled bending DEAs. Adapted with permission from Li (J) et al. (2019). Copyright 2019, Mary Ann Liebert, Inc.

Generally, as illustrated in Figure 1, depending on the material adopted for the DE membrane, the rolled DEAs can be prepared by following two different processes. Figure 1A presents a stepwise fabrication process of a silicone-based rolled DEA. Both Ecoflex and Sylgard, commercially-available polydimethylsiloxane (PDMS), have been widely exploited for the silicone-based DE membrane (Youn et al., 2020). Unlike pre-cured elastomers, silicone elastomers allow thickness control, as they can be solidified at an arbitrary shape because liquid elastomers can maintain chain mobility at room temperature for over an hour, which provides enough time for structural forming *via* bar coating or spin-coating. A single-layered DEA is prepared by curing the thin liquid elastomer, followed by establishing a thin conductive layer on both surfaces and the DEA can be a multi-layered structure by repeating the fabrication process. Particularly, their rolled configuration can be achieved by cutting the membrane DEA into a strip, rolling, and then integrating a couple of rigid covers onto both ends of the structure.

In parallel, 3 M VHB acrylic elastomer-based rolled DEAs can be constructed by following the fabrication process illustrated in Figure 1B. Firstly, two acrylic DE membranes are cut from a 3 M VHB tape batch. Then, the membranes are bi-axially pre-stretched and conductive layers are established. The pre-stretching is done to enhance actuation performance by improving the material’s electromechanical properties, such as its electrical breakdown strength, response time, and pull-in stability (Prabhakar et al., 2020; Sahu et al., 2020; Sahu et al., 2021). Then, the stretched DE membranes are attached. Next, the spring length is matched or compressed to match the width of the brushed electrode pattern. Finally, the spring-rolled DEA is established by rolling the spring structure along the circumferential direction of the stacked DE membrane. With the spring release, the wrapped membrane is further pre-stretched, until the force equilibrium is reached between the compressed spring and the stretched membrane. The deduction in the membrane’s thickness in the fabrication stage further increases the actuator’s linear stroke performance and enhances its breakdown strength, ensuring actuation reliability. (Amin and Assal, 2018; Li et al., 2018).

Applications of the silicone-based DEAs are depicted in Figures 1C–E. The wing flapper shown in Figure 1C is driven by a silicon-based rolled DEA, which is made of BJB TC-5005 silicone as adopting graphite powder for compliant electrodes, and it is reinforced with a shell structure. This structure is bio-inspired from insects’ thoracic mechanism, and it serves to maintain DEA’s pre-stretch, provide external structure, and function as a mechanical amplifier. The prototype dynamically produces a strain of 6.1% at 1 Hz under an electric voltage of 6 kV (Lau et al., 2014). In Figure 1D, the DEA for a micro-aerial vehicle is a combination of a DE membrane based on a silicone mixture of Ecoflex 0030 and Sylgard 184 and a compliant electrode using stamped carbon nanotubes (CNTs). The DEA operates as indirect flight muscles in insects. For its take-off, the vehicle requires a driving frequency of 350 Hz and a voltage of 1.3 kV. At this actuation condition, the resulting strain is around 7% (Chen et al., 2019). In Figure 1E, an array of rolled DEAs is embedded onto a foam-based wearable sleeve in order to communicate and deliver various information to the wearer. The actuator is also composed of a DE membrane based on the aforementioned silicone mixture and a compliant electrode using CNTs. Under 1 kV, the actuator produces strain as high as 12.5% in a frequency ranging from 10 to 200 Hz (Zhao et al., 2020). Compared to acrylic actuators, silicone-based DEAs exhibit high bandwidth, but low to moderate deformation (Chen et al., 2019). Therefore, they are often used in fast-wing flapping motion or vibration applications.

Figures 1F–H depict applications of the acrylic elastomer-based spring-rolled DEAs. Figure 1F shows a walking robot using the spring-rolled DEA (Pei et al., 2003). Here, the bending motion is realized by patterning the electrodes. By activating the front or the back electrode, the DEA is able to bend either forward or backward respectively. Six spring-rolled DEAs, with a maximum bending angle of 60° at 5.9 kV, are attached to a hexagonal frame, and their locomotion was achieved by using a dual tripod gait. Similarly, the DEA in the crawler shown in Figure 1G has patterned electrodes as well. By activating one side, bending is induced and by activating both sides, elongation of the DEA is achieved. The rolled DEA is fabricated with 3 M VHB 4910 with carbon grease as its electrode. At 6.5 kV activation voltage, it shows displacement of 16.5 mm (15% strain) and 108 degrees of bending angle. The locomotion was tested under 1 Hz of actuation frequency, and by integrating with electro-adhesion pads as its feet, it can crawl over inclined surfaces as well (Guo et al., 2022). Figure 1H shows the gripper application of rolled DEAs. It is fabricated from VHB 4910 and patterned carbon grease electrodes. Using the collective bending motion of three rolled DEAs, the gripper’s gripping force is reported as 228.3 mN under a step input voltage of 5 kV (Li J. et al., 2019). As acrylic-based rolled DEAs exhibit larger deformation and higher energy density compared to silicone-based DEAs, they are often integrated with locomotion robots or gripper applications, where large movement or load-bearing capability is of concern rather than its operation bandwidth (Chen et al., 2019).

## 3 Time-dependent modeling of dielectric elastomer muscle

### 3.1 General modeling methods

Since the development of spring-rolled DEA, several modeling approaches have been proposed in order to estimate the electro-mechanical actuation response of DEAs. The modeling approaches can be categorized into two main types, stress-strain models and analytical electromechanical models. In the case of the stress-strain modeling method, the actuation behavior is analyzed by considering the internal physics of the actuator, such as the stress-strain relationship and energy equilibrium within the actuator components. In the beginning stages, the force-actuation stroke relationship of spring-roll DEAs was modeled by considering their geometry. Based on the simulation, it was reported that the output force of the DEA is proportional to its roll stiffness (Pei et al., 2004). Then, a model adopting a stress-strain relationship with an assumption that the output force could be induced by multiple factors including the spring was proposed. As key factors, the elasticity of the materials, pre-strain, and electrostatic force were derived from Young’s modulus, Poisson’s ratio of the material, and the Maxwell stress equation, respectively. Based on the comparison of numerical simulation and experimental results, it was reported that the model could accurately estimate actuation behaviors only for low voltage operations, where the strain is below 4% (Jung et al., 2006). In parallel, another model was developed that formulated a relationship between activation voltage and force output by inserting spring force and electrostatic force terms, which were derived from the Maxwell equation. However, there was a discrepancy between the estimated strain and the achieved strain. Similar to the aforementioned model, the discrepancy tended to increase with activation voltage. To address this issue, they added a correction factor to fit the experimental data (Zhang et al., 2006).

Meanwhile, the analytical electromechanical modeling method considered the step voltage response and frequency response of the DEAs. These responses are analyzed by using conventional electro-mechanical system models, which were expressed as a combination of parameters achieved from experiments. The electrical system could be expressed with the parameters of inductance (L), resistance (R), and capacitance (C). The parameters of mass (m), damping coefficient (c), and spring constant (k) were used for the mechanical system. For an analytical modeling method using the second-order mass-spring-damper model, the model parameters including damped frequency, damping ratio, and damping coefficient are obtained from the step voltage response of the DEA. This work focused on the methodology that could extract system parameters from transient measurements under a fixed voltage level (Tryson et al., 2010). In a wider scope, a dynamic response model formulated by integrating the mechanical mass-spring-damper model with the electrical model composed of R and C was proposed. The expanded analytical model could allow the formulation of static step response as well as frequency dependence in the actuator performance. However, the model requires practical verification and comparison with experimental results (Zhao et al., 2018).

### 3.2 Modeling hyperelasticity under stationary load

As introduced in the previous section, the stress-strain modeling method exhibited limitations in accurately estimating large strain actuation. As the solution, hyperelasticity models have been proposed since the models consider the non-linear nature of dielectric elastomers resulting from long and flexible polymer chains (Moscardo et al., 2008). Various hyperelasticity models have been adopted to estimate the strain energy, such as the neo-Hookean, Gent, Yeoh, and Mooney-Rivlin models (Kovacs et al., 2007; Moscardo et al., 2008; Tryson et al., 2009; Awadalla and Anees, 2011; Sarban et al., 2011; Wang et al., 2016; Amin and Assal, 2018; Li L. et al., 2019). Based on the free energy analysis, Li et al. (2018) proposed a model for the spring-rolled DEA as describing the strain energy of the material with the Gent model. In their work, the actuation performance of three DEAs with different pre-stretch conditions was measured, and model fitting of experimental data was also implemented to obtain their shear modulus and stretch limit. Although the model exhibited consistency with one experimental condition through the whole range of deformation, it rarely showed consistency for different pre-stretch conditions. Recently, another free energy modeling method of the spring-rolled DEA was reported, which describes the strain energy of the material with the Yeoh form. This model was also verified by comparing theoretical data with the experiment (Amin et al., 2020). However, although both hyperelastic modeling approaches contributed to improving modeling capability, the models still could allow accurate estimation of the actuation performance in the specific condition only.

### 3.3 Modeling time-dependency under dynamic load

Although it is reported DEAs are capable of producing electrically-controllable strain, their actuation strain to the applied electric field is non-linear and time-dependent. DEAs also suffer from the viscoelastic creep phenomenon originating from dissipative relaxations within the material. These time-dependent and rate-dependent phenomena are not observed in stationary load conditions, but they prevail under dynamic loading cases. Therefore, addressing the viscoelastic behavior of the DEAs along with hyperelastic modeling is a key factor in improving accuracy in the estimation of their stationary and dynamic actuation performance. In viscoelastic modeling methods, the elastomer could be described by rheological models. As can be seen in the conventional 2-branch general Maxwell model (Zhang J et al., 2015; Li et al., 2021), rheological models are expressed with springs and dampers connected in series and/or in parallel branches. By adding a damper to the system, rheological models could include the rate of deformation to describe the viscoelastic time-dependent relaxation. Through simulation, it was demonstrated that the spring-rolled DEA’s axial stretch undergoes creep deformation with time until the damper is fully relaxed and eventually reaches equilibrium (Zhang C et al., 2015). An expanded rheological model with five parallel branches is also introduced. The model contains the Kelvin-Voigt model with three generalized Maxwell units, which are expressed by a spring and a damper in parallel. The proposed model describes material hyperelasticity through the free energy analysis with the Gent model. Based on experimental comparison among rheological models with different numbers of branches, they verified that the addition of parallel branches to the rheological model improves its prediction capability (Nguyen et al., 2022). However, the verification was limited to the static response of the actuator.

**FIGURE 2:**
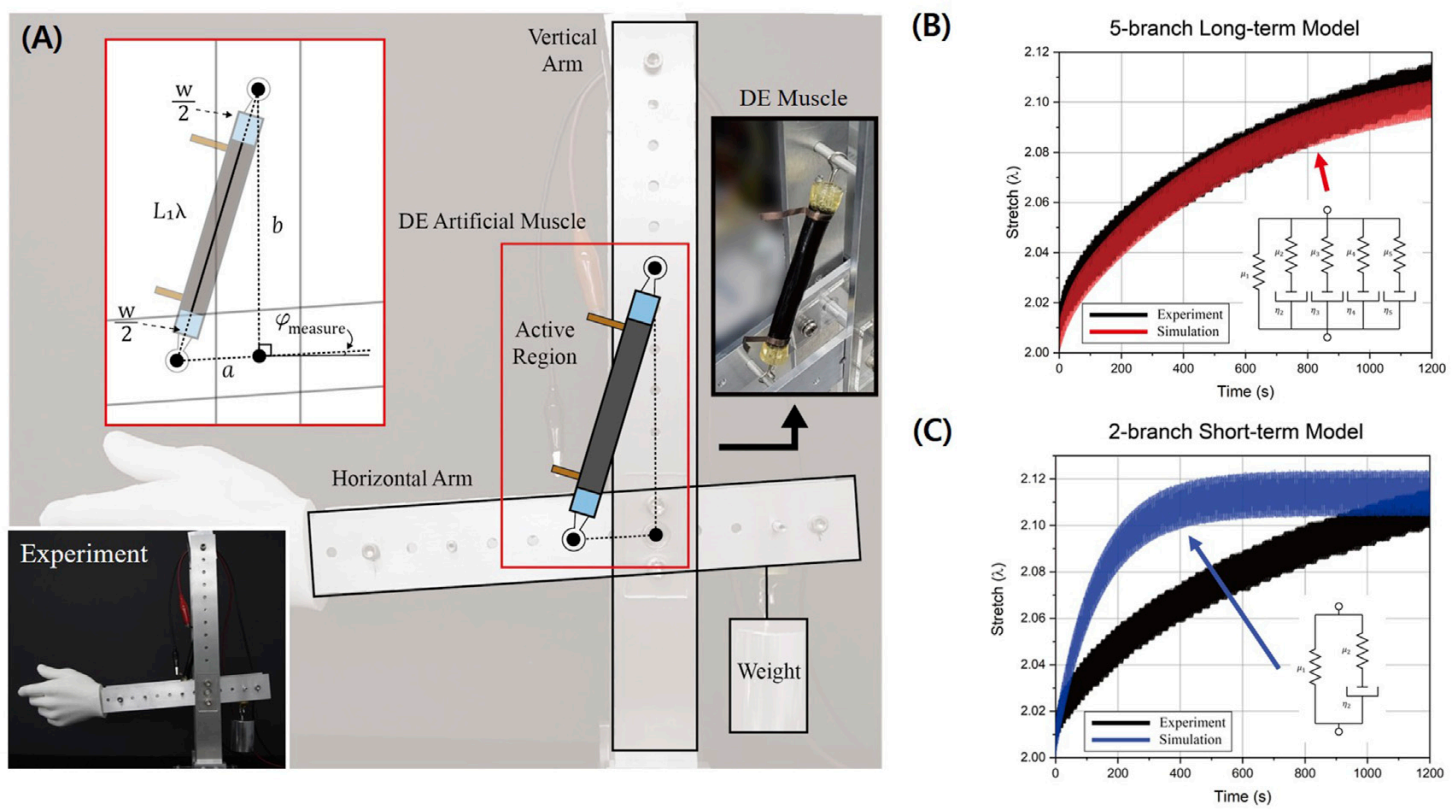
**(A)** Schematic of the DE artificial muscle experimental setup and apparatus. **(B)** Comparison of the long-term 5-branch model simulation (red) and experimental data (black). **(C)** Comparison of the 2-branch model simulation (blue) and experimental data (black).

Meanwhile, in our previous study, we investigated the dynamic behavior of the rolled DEA with a compressed spring core for a period of as long as 20 min (Jeong and Kyung, 2021). Unlike the previous estimation *via* simulation, we found from the observation that viscoelastic creep deformation of the DEA continues even after 20 min of operation, under dynamic voltage input and mechanical loading. Also, we were able to observe rate-dependent behavior, where the actuation stroke decreased with increasing actuation frequency. Considering the creep deformation of the DEA, we established a model that could estimate its long-term dynamic behavior by addressing the behavior with the Gent hyper-elastic model and with the 5-branch rheological model, as shown in the inset of Figure 2B. In this research, the simulated model response followed the experimental result for the entirety of the actuation period of 20 min. Compared with the conventional 2-branch model, it can be seen that the rheological model of a greater number of branches should be utilized for long-term estimation. However, in the previous research, there were 15 unknown parameters, and they had to be model-fit to the experimental data. This requires high computing power and a long time to build the model, which is inadequate for application in practical situations. To address such limitations, amendments are made to the previous model and the improved model is presented below.

$$\begin{array}{c} \frac{k}{L_{\theta }L_{rad}}\left(L_{s}-L_{1}\lambda \right)+\frac{P}{L_{\theta }L_{rad}}\left[\frac{b^{2}+{\left(L_{1}\lambda +w\right)}^{2}-a^{2}}{2b\left(L_{1}\lambda +w\right)}\right]+{\left(\frac{\phi }{L_{rad}}\right)}^{2}\varepsilon \lambda \\ =\mu_{1}\frac{\lambda -\lambda^{-3}}{1-\frac{\lambda^{2}+\lambda^{-2}-2}{J}}+\overset{5}{\underset{i=2}{\sum }}\mu_{i}\frac{\lambda \xi_{i}^{-2}-\lambda^{-3}\xi_{i}^{2}}{1-\frac{\lambda^{2}\xi_{i}^{-2}+\lambda^{-2}\xi_{i}^{2}-2}{J}} \end{array}$$ (2)

$$\begin{array}{c} \frac{d\xi_{i}}{dt}=\frac{\mu_{i}}{\eta_{i}}\frac{\lambda^{2}\xi_{i}^{-3}-\lambda^{-2}\xi_{i}}{1-\frac{\lambda^{2}\xi_{i}^{-2}+\lambda^{-2}\xi_{i}^{2}-2}{J}},for2\leq i\leq 5 \end{array}$$ (3)

Here *k* is the spring constant, *P* is the external load, ϕ is the applied voltage, $L_{s}$ is the initial spring length, $L_{1}$ is the compressed spring length, $L_{\theta }$ is the width of the elastomer, $L_{rad}$ is the thickness of the elastomer, ε is the dielectric constant of the elastomer, λ is the stretch ratio in the axial direction, *J* is the physical stretch limit of the material, $\xi_{i}$ is the stretch ratio of the dashpot component, $\mu_{i}$ is the shear modulus, $\eta_{i}$ is the coefficient of viscosity, and the subscript *i* represents each branch in the rheological model. *a*, *b*, *w* are geometric parameters and they are shown in Figure 2A. Detailed model derivations are included in the Supplementary Material S1.

Further experiments are conducted to verify the proposed model. The spring-rolled DEA is fabricated following the steps outlined in Section 2 using 3 M VHB F9473PC. As the elastomer’s thickness is 254 μm, instead of manually pre-stretching it before rolling, it was pre-stretched after using the compressed spring’s restoration. To observe and verify the estimation capability of the model, a robotic arm assembly was prepared and the DEA was installed to control the arm, as shown in Figure 2A. An encoder was placed at the hinge and the DEA’s deformation was calculated from the angular data. For the experiment, a sinusoidal voltage input of 0.5 Hz frequency and 4000 V amplitude was applied and the actuator’s response was measured for 1,200 s.

The parameters on the left-hand side of the first equation can be obtained from the experimental setup and the fabrication stage, refer to Table 1. $\mu_{1}$ and J can be derived from initial conditions, the initial actuator length, assuming no load is applied to the system. Then, only eight unknown parameters remain, which comprise the time constants of each rheological model branch, ${\tau =\mu }_{i}/\eta_{i}$ . In the model-fit process, in order to minimize computational burden, $\mu_{i}$ and $\eta_{i}$ are assumed to be powers of 10, and $\mu_{i}\leq \eta_{i}$ , so that τ<1 , as the rise time of the actuator response is more than 20 min. For the value optimization, the set of parameters that yields the lowest cost function, compared to the experimental data, is selected. The cost function is composed of root-mean-square error and actuation stroke error. The optimized parameters for the data in Figure 2B are $\mu_{2}=10^{5}$ , $\mu_{3}=10^{5}$ , $\mu_{4}=10^{5}$ , $\mu_{5}=10^{6}$ , $\eta_{2}=10^{7}$ , $\eta_{3}=10^{7}$ , $\eta_{4}=10^{7}$ , $\eta_{5}=10^{7}$ .

**TABLE 1:** Dielectric elastomer muscle fabrication parameters.

Parameter	Value
DE material	3 M VHB F9473PC
Relative permittivity	4.08
Initial DE film dimensions (mm) (*L*_*x*_ *, L*_*y*_ *, L*_*z*_)	60, 250, 0.254
Electrode material	Carbon powder
Electrode dimensions (mm) (*L*_*1*_ *, L*_*2*_)	40, 230
Initial spring length, *L*_*s*_ (mm)	110
Spring diameter (mm)	7
Spring constant, *k* (N/m)	180
Horizontal end point, *a* (mm)	60
Vertical end point, *b* (mm)	110
Passive region length, *w* (mm)	47.3
Applied weight, *P* (g)	72.6
Applied voltage, ϕ (V, Hz)	4000, 0.5
Stretch limit, *J*	200

Figure 2B clearly depicts that the actuator’s relaxation phenomenon (black) is time-dependent and prolongs without reaching an equilibrium when actuated under the dynamic loading condition. The figure verifies that the proposed model addresses both the hyperelasticity and time-dependency of the dielectric elastomer, and as a consequence, it is able to accurately estimate the long-term dynamic response of the actuator. The proposed model’s performance on a different loading condition has been tested and verified as well. Its detailed modeling process and results are included in the Supplementary Material S1. Figure 2C depicts the simulation result of the actuator when the 2-branch rheological model is used. The optimal model parameters are $\mu_{2}=10^{6}$ , and $\eta_{2}=10^{7}$ . It can be observed that for the long-term actuation and under dynamic loading, the conventional 2-branch model is insufficient to accurately model the complex long-term response of the DEA. Therefore, the introduction of multiple branches to the rheological model is necessary, if the DEA were to be operated in practical and long-term applications. As mentioned, the optimization method was selected to minimize the computational burden and the results prove its potential. However, this method has its limitations. For a more accurate estimation of the long-term response, the number of branches in the rheological model can be increased. This in turn increases the number of unknown parameters. As a consequence, despite using the optimization method presented, the computation time for selecting optimal values increases. In Section 4, we further discuss the tradeoff relationship between accuracy and efficiency that follow in the multiple parameter estimation methods.

## 4 Future perspectives and challenges

### 4.1 Artificial intelligence-based data-driven modeling of non-linear response

In the current stress-strain modeling method, the rheological model describing the viscoelasticity of the actuator contains unknown parameters that need to be determined. These parameters are found empirically from experiments. Considering DE muscles’ long-term behavior and their application, the rheological model with multiple branches needs to be used. However, the current parameter optimization process is bound to result in a greater computational burden with the increase in the number of parameters. In addition to the increased burden, the optimization process is prone to fall into local minima, and thus a thorough examination is required.

● *Data-driven modeling*: Considering the complexity of multiple parameter models, approaching the problem with artificial intelligence-based data-driven modeling seems appropriate. Li J. et al. (2019) proposed a model-free control method for DEAs, based on deep reinforcement learning. For the demonstration, simple DEA configurations, circular and rectangular actuators that undergo in-place expansion, are selected and tested. The proposed control method exhibits low trajectory RMSE and shows the potential for the deep learning algorithm to be used in DEAs. For the pursuit of increasing accuracy, the general rolled DEA model proposed in this work could be integrated into the deep learning algorithm. Such integration would allow for the estimation and control of complex structured DEAs, including rolled DEAs. Also, the limitation of increasing the number of branches, and thus unknown parameters, in the rheological model would be overcome. In addition, the study of finding the optimal rheological model for dielectric elastomers could follow.

● *Real-time multiple parameter estimation with computation efficiency*: Increasing the accuracy of the model is of great issue. However, as can be seen in experiments by Tryson et al. (2009), DE actuator’s performance varies per sample. Actuators fabricated in the same fashion do not yield the same results. Moreover, the physical properties of elastomers, such as inertia distribution, viscosity, and stiffness, also vary under dynamic deformation and environmental factors. Due to the variance caused by the coupling of multiple parameters, the modeling and parameter optimization process needs to be carried out per sample for an accurate estimation. In addition, the deep learning method may yield high accuracy, but at the cost of a long model training time. For the model to be used in practical applications, a real-time estimation method needs to be established. An example of a real-time estimation can be witnessed in the haptics field. A data-driven haptic rendering of viscoelastic deformable objects, including Ecoflex silicone is proposed. A random forest regression method was utilized, and the relationship between force and displacement was trained. As a result, the viscoelastic behavior was replicated in the relationship (Bhardwaj et al., 2019). In haptic applications, high perceptual realism is important. Therefore, instead of applying complex physics models and obtaining high accuracy, similarity is pursued and computational efficiency is achieved. As a result, the model is capable of handling large and complex datasets with good accuracy, and at an update rate above 1 kHz, which is sufficient for real-time computation. Such an approach for similarity could also be integrated into the field of real-time DEA modeling and could become an effective and robust solution for actuator control.

### 4.2 Practical considerations for application

Owing to their actuation capability, producing a large and controllable electrically-induced strain with fast response speed, and soft and lightweight configuration, DEAs have been given great attention as a promising candidate for artificial muscle. Advances in DEA technology with respect to material and structural design could contribute to eliciting outstanding actuation performance on par with or exceeding that of a natural muscle. Moreover, the recent development of rolled DEAs opened up opportunities for exploiting them for practical applications since DE muscles could produce much larger force and energy output than conventional membrane DEAs under the same electric voltage.

However, there are still technical challenges in several aspects. In fabrication, multi-layered stacking for most silicone elastomers requires a complicated process, superposing each membrane DEA while suffering from low yield and non-uniformity of layers resulting from curing the liquid resin cast. Operating voltage for the DEAs is also limited due to electro-mechanical instability (EMI) impeding recovery from a deformed state, electrical safety for human contact, and the necessity of a compact power source for miniaturization of the control system. Particularly, for practical use in the fields of robotics, tactile display, and human-assistive devices, the following aspects need to be considered further.

• *Robotic kinematic application*: DE muscles have been utilized to operate robotic systems. A robotic hand, where DE muscles move fingers through a pulley mechanism (Jung et al., 2006), and a cable-driven hand exoskeleton for rehabilitation, where DE muscles assist finger extension (Amin et al., 2020), have been proposed. In such robotic kinematic applications, it is important to amplify the DE muscle’s mechanical strength and/or its displacement. For the amplification, adopting a multi-layered DE structure and increasing the actuation voltage may be required to improve its performance. Externally, the actuator can be reinforced with exoskeletons, that can serve as mechanical amplifiers. In addition, the actuator’s durability and robustness upon long-time dynamic loading need to be guaranteed.

• *Tactile/haptic display*: Proposed tactile or haptic feedback devices include the force feedback glove (Zhang et al., 2006), Braille displays (Ren et al., 2008; Levard et al., 2011), and haptic sleeve (Zhao et al., 2020). In tactile displays, their actuation bandwidth and wearability, including safety, are major factors that require attention. As humans are sensitive to stimulations up to 200 Hz, elastomer material needs to be selected considering its frequency response. Thus, silicone-based elastomers are favorable among various material candidates. In addition, for the device to be wearable, its circuitry needs to be compact and wireless. Therefore, work on miniaturizing and insulating the high-voltage circuit is necessary. For safety, it is recommended to use either thin-film elastomers to lower the DE muscle’s operation voltage (Ji et al., 2020), embed wiring within the device, or insulate contact points with an additional elastomer layer.

• *Bio-inspired robot*: DE muscles are frequently used for soft robots, as their actuation resembles the motions of nature and animals. Several designs have been proposed, ranging from locomotion robots mimicking inchworms (Wang et al., 2016; Li L. et al., 2019; Guo et al., 2022), to eyeball muscles for humanoid robot control (Li et al., 2018), and to flying robots inspired from insect wings (Lau et al., 2014; Chen et al., 2019). These robots require either fast movement or large force output. For a wider bandwidth, a more elastic material, such as silicones, should be selected. If its force exertion is inadequate, it can be enhanced through mechanical amplifiers, stacking, or arranging actuators in an array. Similar to tactile display, ultimately for their free-roaming, methods to develop untethered robots should be further investigated. In addition, most reported applications operate between two states. That is on and off. As proposed in previous sections, an accurate model needs to be developed to control these robots.

## Data Availability

The raw data supporting the conclusion of this article will be made available by the authors, without undue reservation.
