# Supplementary material for: Modeling dynamic behavior of dielectric elastomer muscle for robotic applications
Source: Front Bioeng Biotechnol. 2023 Feb 10;11:1006346. doi: 10.3389/fbioe.2023.1006346 (PMC9950498; doi:10.3389/fbioe.2023.1006346)
Supplement: Supplementary file 1 [file DataSheet1.DOCX]

Supplementary Material

# Modeling of the Dielectric Elastomer Muscle

The dielectric elastomer muscle is modeled based on the free energy analysis of the actuator system. For a dielectric elastomer actuator to deform from one state to another, energy needs to be input into the system. Such a relationship can be expressed with the Helmholtz free energy equation.

$$\begin{aligned} W=W_{s}+\frac{D^{2}}{2\varepsilon} \#\left( 1 \right) \end{aligned}$$

The term on the left side represents the Helmholtz free energy applied to the dielectric elastomer actuator. The first term on the right side shows the energy associated with the stretching of the elastomer, and the second term represents the electrostatic energy of the film, where D is the electrical displacement and $\varepsilon$ is the dielectric constant or permittivity of the material.

For the artificial muscle, a cylindrical actuator is fabricated by wrapping a thin film around a spring core. As the film is adhesive, it can be assumed that the actuator does not deform in the circumferential direction upon voltage application, due to its strong adhesion between adjacent layers. Defining the stretch ratio as $\lambda=l/L$, where L represents the initial length, and *l* represents the measured length, the circumferential stretch ratio, $\lambda_{\theta}$, can be assumed as:

$$\begin{aligned} \lambda_{\theta}=\frac{l_{\theta}}{L_{\theta}}=1 \#\left( 2 \right) \end{aligned}$$

Assuming the elastomer is incompressible, and using cylindrical coordinates where 1, $\theta$, and rad denote axial, circumferential, and radial directions respectively,

$$\begin{aligned} \lambda_{1}\lambda_{\theta}\lambda_{rad}=1 \#\left( 3 \right) \end{aligned}$$

Since the fabricated muscle actuates in the axial direction,

$$\begin{aligned} \lambda_{1}=\lambda\#\left( 4 \right) \end{aligned}$$

$$\begin{aligned} \lambda_{rad}=\frac{1}{\lambda} \#\left( 5 \right) \end{aligned}$$

Then the Helmholtz free energy term related to external work applied to the actuator can be expressed as the following:

$$\begin{aligned} L_{1}L_{\theta}L_{rad}\delta W=\phi\delta Q+k\left( L_{s}-L_{1}\lambda\right)L_{1}\delta\lambda+P\left[ \frac{b^{2}+\left( L_{1}\lambda+w \right)^{2}-a^{2}}{2b\left( L_{1}\lambda+w \right)} \right]L_{1}\delta\lambda\#\left( 6 \right) \end{aligned}$$

Here $\phi$ is the applied voltage, Q is the charge, k is the compression spring’s spring constant, L_s_ is the spring’s initial length, L_1_ is the compressed spring’s length and compliant electrode’s width,$L_{\theta}$ is the circumferential length of the wrapped film which is the same as L_y_, L_rad_ is the thickness of the film which is the same as L_z_, P is applied mechanical load, a and b are muscle’s end position distances from the robot arm hinge joint, and w represents the length of the artificial muscle that does not actuate. The three terms on the right side represent the electrical, spring, and mechanical work done to stretch the dielectric elastomer. Introducing electrical displacement, $D=\frac{Q}{L_{1}L_{\theta}\lambda}$, and differentiating the left side of equation 6 with respect to stretch ratio and electrical displacement, the following equations are derived.

$$\begin{aligned} \frac{\delta W}{\delta\lambda}=\frac{\phi}{L_{rad}}D+\frac{k}{L_{\theta}L_{rad}}\left( L_{s}-L_{1}\lambda\right)+\frac{P}{L_{\theta}L_{rad}}\left[ \frac{b^{2}+\left( L_{1}\lambda+w \right)^{2}-a^{2}}{2b\left( L_{1}\lambda+w \right)} \right] \#\left( 7 \right) \end{aligned}$$

$$\begin{aligned} \frac{\delta W}{\delta D}=\frac{\phi}{L_{rad}}\lambda\#\left( 8 \right) \end{aligned}$$

The work related to stretching of the elastomer is represented with the Gent hyper-elastic model and the 5-branch rheological model, shown in the subset of Figure 2B. Considering the rheological model, upon elastomer stretch, five branches elongate together at a ratio of $\lambda$. Defining the stretch ratio of the spring component as $\lambda^{e}$ and the dashpot component as $\xi$, then $\lambda$ of each branch is expressed as:

Elastic branch: $\lambda=\lambda^{e}$ and Viscoelastic branch: $\lambda=\lambda^{e}\xi$

Using the above expressions and the Gent model, the stretch work, W_s_, is derived.

$$\begin{aligned} W_{s}=-\frac{\mu_{1}J_{1}}{2}\ln\left( 1-\frac{\lambda_{1}^{2}+\lambda_{\theta}^{2}+\lambda_{rad}^{2}-3}{J_{1}} \right)-\sum_{i=2}^{5} \frac{\mu_{i}J_{i}}{2}\ln\left( 1-\frac{\lambda_{1}^{2}\xi_{i1}^{-2}+\lambda_{\theta}^{2}\xi_{i\theta}^{-2}+\lambda_{rad}^{2}\xi_{i rad}^{-2}-3}{J_{i}} \right) \#\left( 9 \right) \end{aligned}$$

Here J represents the physical stretch limit of the elastomer and $\mu_{i}$ represents the shear modulus of each branch in the rheological model. Since branches are connected in parallel, their stretch limits are assumed to be equal, $J_{i}=J$ for all *i*. Assuming incompressibility and no elongation in the circumferential direction, equation 9 can be simplified. Combining with the right side of equation 1:

$$\begin{aligned} W=-\frac{\mu_{1}J}{2}\ln\left( 1-\frac{\lambda^{2}+\lambda^{-2}-2}{J} \right)-\sum_{i=2}^{5} \frac{\mu_{i}J}{2}\ln\left( 1-\frac{\lambda^{2}\xi_{i}^{-2}+\lambda^{-2}\xi_{i}^{2}-2}{J} \right)+\frac{D^{2}}{2\varepsilon} \#\left( 10 \right) \end{aligned}$$

Differentiating with respect to stretch ratio and electric displacement:

$$\begin{aligned} \frac{\delta W}{\delta\lambda}=\mu_{1}\frac{\lambda-\lambda^{-3}}{1-\frac{\lambda^{2}+\lambda^{-2}-2}{J}}+\sum_{i=2}^{5} \mu_{i}\frac{\lambda\xi_{i}^{-2}-\lambda^{-3}\xi_{i}^{2}}{1-\frac{\lambda^{2}\xi_{i}^{-2}+\lambda^{-2}\xi_{i}^{2}-2}{J}} \#\left( 11 \right) \end{aligned}$$

$$\begin{aligned} \frac{\delta W}{\delta D}=\frac{D}{\varepsilon} \#\left( 12 \right) \end{aligned}$$

Rearranging equations 7, 8, 11, and 12,

$$\begin{aligned} D=\frac{\phi}{L_{rad}}\varepsilon\lambda\#\left( 13 \right) \end{aligned}$$

$$\begin{aligned} \frac{k}{L_{\theta}L_{rad}}\left( L_{s}-L_{1}\lambda\right)+\frac{P}{L_{\theta}L_{rad}}\left[ \frac{b^{2}+\left( L_{1}\lambda+w \right)^{2}-a^{2}}{2b\left( L_{1}\lambda+w \right)} \right]+\left( \frac{\phi}{L_{rad}} \right)^{2}\varepsilon\lambda\\ =\mu_{1}\frac{\lambda-\lambda^{-3}}{1-\frac{\lambda^{2}+\lambda^{-2}-2}{J}}+\sum_{i=2}^{5} \mu_{i}\frac{\lambda\xi_{i}^{-2}-\lambda^{-3}\xi_{i}^{2}}{1-\frac{\lambda^{2}\xi_{i}^{-2}+\lambda^{-2}\xi_{i}^{2}-2}{J}} \#\left( 14 \right) \end{aligned}$$

To model the time-dependent response of the actuator as well, the following stretch rate relationship is used:

$$\begin{aligned} \frac{d\xi_{i}}{dt}=-\frac{1}{\eta_{i}}\frac{\partial W}{\partial\xi_{i}} \#\left( 15 \right) \end{aligned}$$

$$\begin{aligned} \frac{d\xi_{i}}{dt}=\frac{\mu_{i}}{\eta_{i}}\frac{\lambda^{2}\xi_{i}^{-3}-\lambda^{-2}\xi_{i}}{1-\frac{\lambda^{2}\xi_{i}^{-2}+\lambda^{-2}\xi_{i}^{2}-2}{J}} \mathrm{for} 2\leq i\leq5 \#\left( 16 \right) \end{aligned}$$

Here $\eta_{i}$ is the coefficient of viscosity. It can be seen that $\mu_{i}$ and $\eta_{i}$ form pairs and by controlling their ratio, the rise time of the response can be modulated. Thus, their ratio, $\tau_{i}=\mu_{i}/\eta_{i}$, can be considered as the *i*^th^ viscoelastic branch’s time constant. Therefore, the set of one stretch equation and four stretch rate equations forms the general model of a dielectric elastomer muscle. It is reproduced below.

$$\begin{aligned} \frac{k}{L_{\theta}L_{rad}}\left( L_{s}-L_{1}\lambda\right)+\frac{P}{L_{\theta}L_{rad}}\left[ \frac{b^{2}+\left( L_{1}\lambda+w \right)^{2}-a^{2}}{2b\left( L_{1}\lambda+w \right)} \right]+\left( \frac{\phi}{L_{rad}} \right)^{2}\varepsilon\lambda\\ =\mu_{1}\frac{\lambda-\lambda^{-3}}{1-\frac{\lambda^{2}+\lambda^{-2}-2}{J}}+\sum_{i=2}^{5} \mu_{i}\frac{\lambda\xi_{i}^{-2}-\lambda^{-3}\xi_{i}^{2}}{1-\frac{\lambda^{2}\xi_{i}^{-2}+\lambda^{-2}\xi_{i}^{2}-2}{J}} \#\left( 17 \right) \end{aligned}$$

$$\begin{aligned} \frac{d\xi_{2}}{dt}=\frac{\mu_{2}}{\eta_{2}}\frac{\lambda^{2}\xi_{2}^{-3}-\lambda^{-2}\xi_{2}}{1-\frac{\lambda^{2}\xi_{2}^{-2}+\lambda^{-2}\xi_{2}^{2}-2}{J}} \#\left( 18 \right) \end{aligned}$$

$$\begin{aligned} \frac{d\xi_{3}}{dt}=\frac{\mu_{3}}{\eta_{3}}\frac{\lambda^{2}\xi_{3}^{-3}-\lambda^{-2}\xi_{3}}{1-\frac{\lambda^{2}\xi_{3}^{-2}+\lambda^{-2}\xi_{3}^{2}-2}{J}} \#\left( 19 \right) \end{aligned}$$

$$\begin{aligned} \frac{d\xi_{4}}{dt}=\frac{\mu_{4}}{\eta_{4}}\frac{\lambda^{2}\xi_{4}^{-3}-\lambda^{-2}\xi_{4}}{1-\frac{\lambda^{2}\xi_{4}^{-2}+\lambda^{-2}\xi_{4}^{2}-2}{J}} \#\left( 20 \right) \end{aligned}$$

$$\begin{aligned} \frac{d\xi_{5}}{dt}=\frac{\mu_{5}}{\eta_{5}}\frac{\lambda^{2}\xi_{5}^{-3}-\lambda^{-2}\xi_{5}}{1-\frac{\lambda^{2}\xi_{5}^{-2}+\lambda^{-2}\xi_{5}^{2}-2}{J}} \#\left( 21 \right) \end{aligned}$$

# Parameter Optimization

The general model, proposed previously in equations 17-21, is composed of various parameters. Parameters on the left-hand side of equation 17 can be obtained from the experimental environment and from the fabrication stage. However, parameters on the right-hand side of equation 17 and in stretch rate equations remain unknown. A total of 10 unknown parameters exist and they are $\mu_{i}, where 1\leq i\leq5$, $\eta_{j}, where 2\leq j\leq5$, and J. Among unknown parameters, two values can be derived from boundary conditions.

In the fabrication process, when the locking mechanism is removed from the rolled dielectric elastomer muscle, the muscle elongates solely due to the restoration force of the compression spring. As the elongation is completed, it can be assumed that equilibrium has been reached in the DE muscle. Then, as $t\to\infty$, $\frac{d\xi_{i}}{dt}\to0$ and $\xi_{i}\to\lambda$. At this equilibrium state, neither mechanical load nor voltage is applied to the muscle. As a result, the general equation is simplified as the following.

$$\begin{aligned} \frac{k}{L_{\theta}L_{rad}}\left( L_{s}-L_{1}\lambda\right)=\mu_{1}\frac{\lambda-\lambda^{-3}}{1-\frac{\lambda^{2}+\lambda^{-2}-2}{J}} \#\left( 22 \right) \end{aligned}$$

Using the experimental condition shown in Table 1 of the manuscript and setting $\lambda=2$, equation 22 is reduced to the relationship between $\mu_{1}$ and J. The value of $\mu_{1}$ is dependent on the stretch limit, J, and becomes positive when $J>\lambda^{2}+\lambda^{-2}-2$. Also, $\mu_{1}$ converges to a value when $J>100$.

The elastomer’s stretch limit, J, can be calculated by measuring the DE’s stretch under uniaxial tension.

$$\begin{aligned} J=\lambda_{limit}^{2}+\lambda_{limit}^{-2}-2 \#\left( 23 \right) \end{aligned}$$

The VHB tape can be stretched up to 15 to 16 times its original length. Applying equation 23, the value of J is estimated to be between 223 to 254. However, since $\mu_{1}$ converges when $J>100$, the stretch limit is set conservatively as 200. As a result, the parameters J and $\mu_{1}$ are obtained from experimental conditions, and unknown parameters are now reduced to eight parameters, that are related to the four viscoelastic branches of the rheological model.

The remaining unknown parameters comprise four time constants of viscoelastic branches. Parameters, shear moduli, and viscosity coefficients, are determined by model-fitting the experimental data. For the model-fitting process, two objective functions are used to optimize parameters, the root-mean-square error and the average terminal stroke error. For optimization, an arbitrary value is assigned to each unknown parameter, and the corresponding actuation response is simulated. Then, the response is evaluated using the two objective functions. The set of parameter values that minimize the combined cost is chosen as the optimal parameter set. As there may exist an infinite number of possible parameter combinations, the input domain for shear moduli and viscosity coefficients is bound to minimize the number of computations. According to VHB tape’s datasheet, its shear strength is reported to be 550 kPa (3M, 2021). Thus, $\mu_{i}$ are bound between 10^4^ and 10^6^ Pa. Referring to Figure 2, it can be seen that the time-dependent drifting phenomenon continues even after 1000 seconds of actuation. This infers that there is a possibility that the time constant of an arbitrary viscoelastic branch may reach up to 10^-3^. As a result, $\eta_{j}$ are bound between 10^4^ and 10^10^. The step interval for the input domain is set as powers of 10. Then, the input domain for unknown parameters is: $\mu_{i}=\left\{ {10}^{4},{10}^{5},{10}^{6} \right\}, \eta_{j}=\{{10}^{4},{10}^{5},{10}^{6},{10}^{7},{10}^{8},{10}^{9},{10}^{10}\}$. Simulation responses are obtained by solving the derived equation set in MATLAB using the ode15s function. Each simulation’s root-mean-square error is calculated by comparing it to the experimental data. In addition, another objective function, the terminal actuation stroke error is calculated. The terminal actuation stroke error is calculated by comparing the last 10 seconds of the response data.

Finally, the condition with the lowest terminal stroke error is selected as the optimal parameter combination. Supplementary Figure 1A shows the response of the 5-branch model with optimal conditions for the 0.5 Hz actuation condition. Selected parameters are $\mu_{2}={10}^{5},\eta_{2}={10}^{7}, \mu_{3}={10}^{5}, \eta_{3}={10}^{7}, \mu_{4}={10}^{5}, \eta_{4}={10}^{7}, \mu_{5}={10}^{6}, \eta_{5}={10}^{7}$. The resulting RMS error is $7.011*{10}^{-3}$, and the terminal stroke error is 5.792%. Supplementary Figure 1B shows the actual response of the 0.25 Hz dynamic loading case, in black, and the simulated response of the dielectric elastomer muscle, in orange. At a slower loading rate, the DE muscle displays increased stroke compared to the 0.5 Hz actuation case. The same modeling process is applied and the results verify its ability to predict different loading conditions. Selected parameters are $\mu_{2}={10}^{4},\eta_{2}={10}^{7}, \mu_{3}={10}^{4}, \eta_{3}={10}^{10}, \mu_{4}={10}^{4}, \eta_{4}={10}^{8}, \mu_{5}={10}^{4}, \eta_{5}={10}^{7}$. The resulting RMS error is $94.50*{10}^{-3}$, and the terminal stroke error is 10.66%. It can be observed from the figure that the optimized model is able to accurately estimate the long-term response trend of a dielectric elastomer muscle under both electric and mechanical dynamic loading.

**
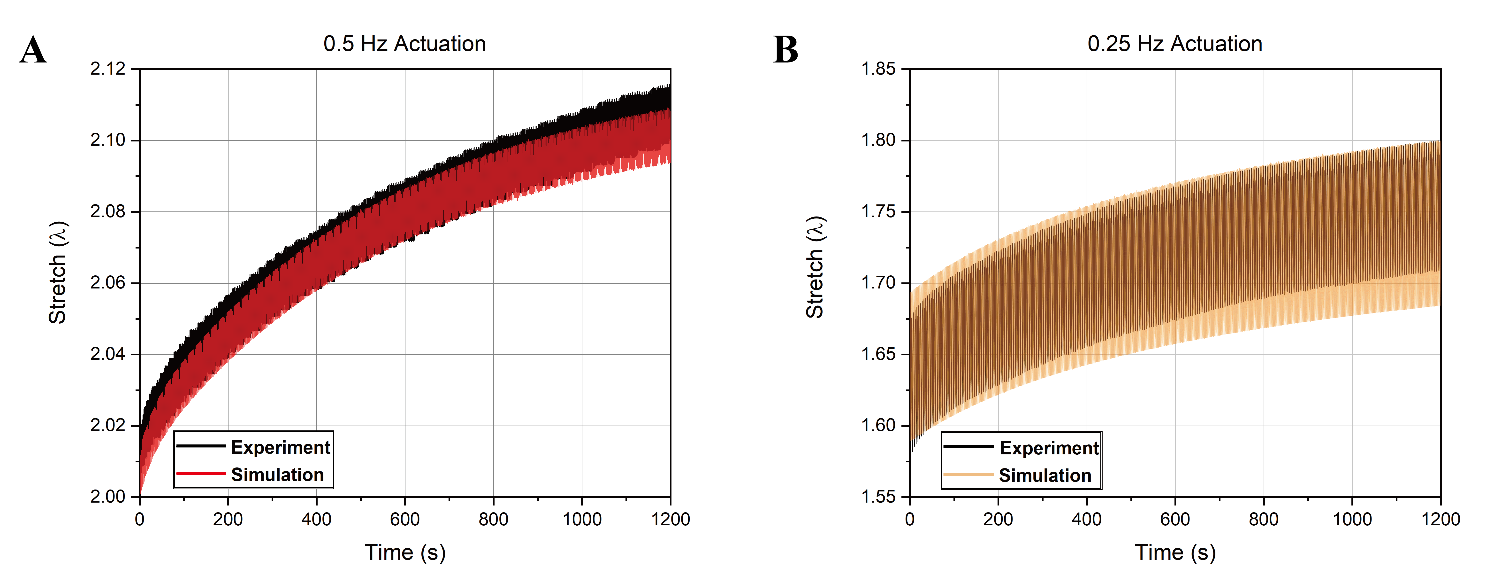
**

**Supplementary Figure 1.** Actuation responses of dielectric elastomer muscle under different loading conditions. (**A**) Experimental (black) and simulated (red) responses of the DE muscle under 0.5 Hz electrical loading. (**B**) Experimental (black) and simulated (orange) responses of the DE muscle under 0.25 Hz electrical loading.
